# Cytokines in the Immune Microenvironment Change the Glycosylation of IgG by Regulating Intracellular Glycosyltransferases

**DOI:** 10.3389/fimmu.2021.724379

**Published:** 2022-01-24

**Authors:** Yedi Cao, Zhijing Song, Zhendong Guo, Xue Zhao, Yan Gong, Keli Zhao, Chenxue Qu, Youyuan Huang, Yan Li, Ying Gao, Junqing Zhang, Xiaohui Guo

**Affiliations:** ^1^ Department of Endocrinology, Peking University First Hospital, Beijing, China; ^2^ Key Laboratory of Interdisciplinary Research, Institute of Biophysics, Chinese Academy of Sciences, Beijing, China; ^3^ College of Life Science, University of Chinese Academy of Sciences, Beijing, China; ^4^ Department of Clinical Laboratory, Peking University First Hospital, Beijing, China

**Keywords:** IgG, glycosylation, cytokines, B cell, glycosyltransferase, autoimmune diseases

## Abstract

**Background:**

Changes in IgG glycosylation, as a novel pathological feature, are observed in various autoimmune diseases (AIDs). The glycosylation patterns of IgG play a critical role in regulating the biological function and stability of IgG involved in the pathophysiology of many AIDs. However, the intracellular regulatory mechanisms underlying the effects of disturbances in various cytokines on IgG glycosylation are poorly understood. Thus, we investigated the regulatory effects of elevated cytokines in AIDs on intracellular IgG glycosylation within B cells.

**Methods:**

First, we established a controlled primary culture system *in vitro* to differentiate human CD19^+^ B cells into antibody-secreting cells (ASCs). Then, the IgG concentrations in the supernatants were measured by enzyme-linked immunoassay (ELISA) under IFN-γ, TNF-α, IL-21, IL-17A, BAFF, or APRIL stimulation. Next, the glycosylation levels of IgG under different stimuli were compared *via* a lectin microarray. The fine carbohydrate structures of IgG were confirmed by matrix-assisted laser desorption/ionization-quadrupole ion trap-time of flight-mass spectrometry (MALDI-TOF-MS). Finally, the expression of glycosyltransferases and glycosidases in B cells under stimulation with several cytokines was detected by real-time PCR and western blotting.

**Results:**

We found that cytokines significantly promoted IgG production *in vitro* and led to considerably different IgG glycan patterns. Specifically, the results of lectin microarray showed the galactose level of IgG was increased by IFN-γ stimulation (*p*<0.05), and the sialylation of IgG was increased by IL-21 and IL-17A (*p*<0.05). The MALDI-TOF-MS data showed that the frequency of agalactosylation was decreased by IFN-γ with the increased frequency of mono-galactosylation and decreased frequency of digalactosylation, accompanied by upregulation of β-1,4-galactosyltransferase 1. Both frequencies of mono-sialylated and disialylated N-glycans were increased by IL-21 and IL-17A with decreased frequency of asialylation, and the expression of β-galactoside α-2,6-sialyltransferase 1 was upregulated by IL-21 and IL-17A.

**Conclusion:**

Abnormally elevated cytokines in the microenvironment regulates IgG glycan patterns by regulating intracellular glycosyltransferases in human B cells.

## Introduction

The appearance of autoantibodies in circulation, which are mainly IgG, is a characteristic of autoimmune diseases (AIDs). Autoantibodies can mediate immune responses against autoantigens, leading to inflammation and destruction by antibody-dependent cell-mediated cytotoxicity (ADCC) and complement-dependent cytotoxicity (CDC) (1–3).

**Figure 1:**
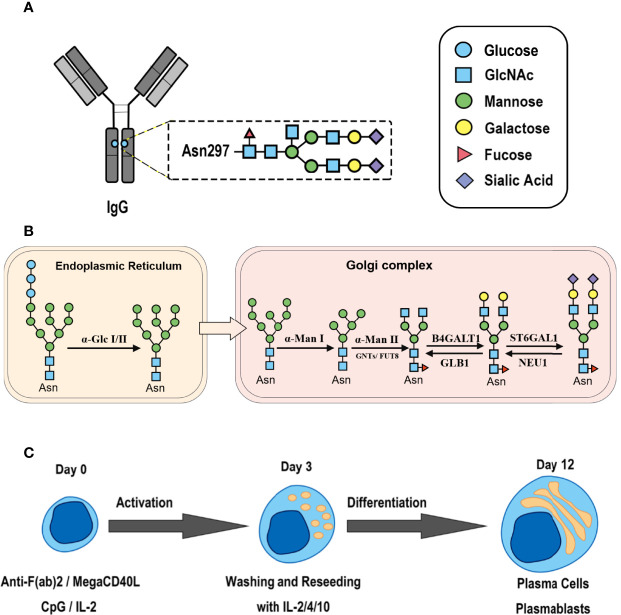
Synthesis of N-glycan in IgG and the two-step differentiation system of B cells. **(A)** Conserved repertoire of an N-linked glycan attached to the Fc domain of IgG at Asn297, which has a biantennary core heptasaccharide consisting of a chain with two N-acetylglucosamines (GlcNAc) and a mannose, followed by two mannose branches and a further GlcNAc following each mannose. The optional residues of a core fucose, a bisecting GlcNAc, one or two galactoses, and sialic acids can attach to the core structures to enrich the structural diversity of IgG. **(B)** Sythesis of N-glycan in B cells. Various α-mannosyltransferases (ALGs) catalyze the synthesis of triantennary Glc_3_Man_9_GlcNAc_2_ glycans in the lumen of the endoplasmic reticulum (ER). Then, α-glucosidases I and II remove α-glucose from the sugar chain to form the high-mannose type Glc_3_Man_5~9_. After transfer into the Golgi complex, α-mannosidase I trims mannose residues from the N-glycan to form Man_5_GlcNAc_2_, which is the core structure of hybrid-type N-glycans. Next, α-mannosidase II removes the two α-mannoses from the glycan chain, and two GlcNAc sequences are catalyzed by N-acetylglucosaminyltransferases (GNTs) to form the biantennary heptasaccharide core structure Man_3_GlcNAc_4_. Then, α-1,6-fucosyltransferase 8 (FUT8) catalyzes the addition of fucose to the core structure. β-1,4-Galactosyltransferase 1 (B4GALT1) and β-galactosidase (GLB1) are responsible for the addition and removal of galactose. β-Galactoside α-2,6-sialyltransferase 1 (ST6GAL1) and sialidase-1 (NEU1) transfer and cleave sialic acid to/from oligosaccharides. **(C)** B cell *in vitro* differentiation system. First, B cells were activated by anti-F(ab’)_2_, MegaCD40 L, CpG ODN, and IL-2 for 3 days and then washed and reseeded with IL-2, IL-4, and IL-10 to help activate B cell differentiation into antibody-secreting cells (ASCs) for up to 12 days.

IgG is a glycoprotein secreted by antibody-secreting cells (ASCs) and is composed of a crystallizable fragment (Fc) responsible for triggering effector functions and Fab arms responsible for antigen binding. Two conserved repertoires of N-linked glycans, each composed of a biantennary heptasaccharide core structure and optional terminal glycans, are attached to the Fc tail *via* the asparagine residue at position 297 (Asn297) (**Figure 1A**). Glycosylation patterns differ between the Fab and Fc regions, and the former contains glycans with high sialylation (up to 93%) (4). The glycan patterns of IgG vary widely among different immune states (5, 6), and they expand the functional repertoires of IgG. In the literature, the level of IgG glycans lacking a galactose residue (G0-IgG) is remarkably correlated with the disease activity of rheumatoid arthritis (RA) (7, 8). In our previous study, we found that the glycosylation levels of TgAb IgG were increased in patients with Hashimoto’s thyroiditis (HT) compared to healthy donors (9, 10). Glycan patterns are not templated but remarkably dynamic and govern the biological functions of IgG by affecting its affinity to Fcγ receptors and C1q (11–14), leading to a wide range of immune responses (15). Therefore, carbohydrate structures are critical for modulating the biological functions of IgG in the execution phase of the immune response, and an investigation of the mechanisms underlying the effects of changes in IgG glycosylation could shed new light on the pathogenesis and progression of AIDs.

As shown in **Figure 1B**, the processing pathway of the N-linked glycan structure occurs in a strictly sequential manner by two major enzyme families, namely, glycosyltransferases and glycosylhydrolases (16). It has been reported that multiple factors, such as interleukin-21 (17, 18), antigens (19), nucleotide sugar precursors in culture medium (20), and activated platelets (21), are involved in dynamically regulating the glycosylation of IgG. However, studies on the regulatory mechanism of glycosylation have mainly focused on recombinant IgG in antibody-producing cell lines based on genetic engineering (22, 23) and extracellular modification of serum IgG treated with soluble enzymes (24, 25), and little is known about the effects of cytokines in the microenvironment on changes in the IgG glycan profile and glycosylation enzymes during the differentiation of B lymphocytes.

Elevations of various cytokines in both circulation and the site of inflammation have been reported in AIDs (15, 26). An imbalance between T helper (Th)1 cells/Th2 cells and Th17 cells/Treg cells was reported to play a critical role in the breakdown of immune tolerance and prompt the development of AIDs with local production of cytokines (including IFN-γ, TNF-α, IL-21, and IL-17A), including RA, systemic lupus erythematosus (SLE), Sjogren’s syndrome (SS), and autoimmune thyroid diseases (AITD) (27–32). In addition, B-cell-activating factor (BAFF, formerly BLyS) and a proliferation-inducing ligand (APRIL) were reported to be overexpressed in AIDs, which is a survival tactic that supports autoreactive B cells and may represent a critical event that disrupts the immune tolerance of B cells (33–36). The contributions of these elevated cytokines to IgG glycosylation and glycan processing enzymes in B cells remains poorly characterized.

Thus, we established a two-step *in vitro* B cell differentiation system and explored whether various cytokines commonly elevated in AIDs contribute to changes in the IgG glycosylation pattern by regulating the expression of intracellular glycosylation enzymes in B cells.

## Methods

### Two-Step *In Vitro* B Cell Differentiation System

This study complied with the Declaration of Helsinki, was approved by the Medical Ethics Committee of Peking University First Hospital and was conducted in accordance with approved guidelines. All participants provided written informed consent (2021–318).

**Figure 2:**
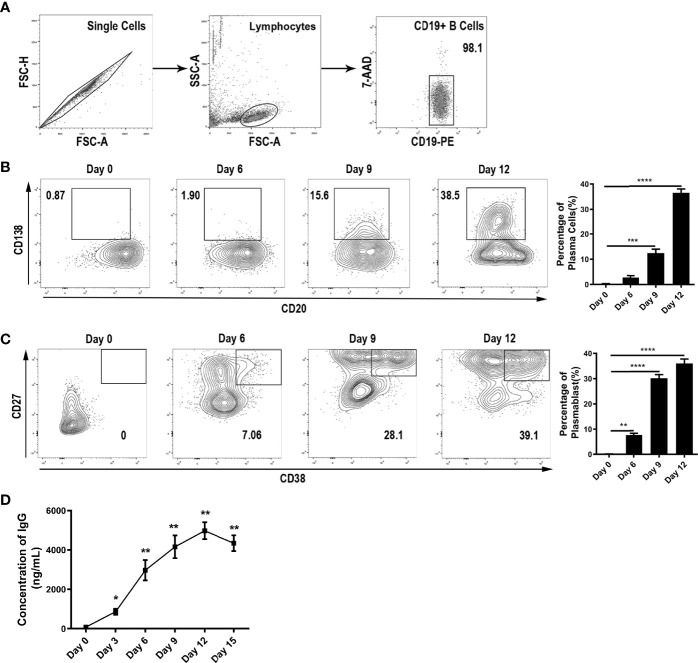
B cell differentiation into ASCs *in vitro*. **(A)** Purity of B cells after sorting by immunomagnetic beads. **(B–C)** Differentiation of B cells into ASCs was monitored by flow cytometry *via* the surface expression of CD138, CD20, CD27, and CD38 on Day 0, Day 6, Day 9, and Day 12. All events were gated by the 7-AAD^neg^ population. The population of CD138^+^CD20^low^ represented plasma cells, and the CD27^high^CD38^+^ population represented plasmablasts. The bar charts shown on the right indicate the percentage of ASCs along with the stimulation time. The error bars represent the SD. *p < 0.05, **p < 0.001, ***p < 0.0001, ****p < 0.00001. **(D)** Measurement of IgG in the culture medium was carried out continuously for up to 15 days. The error bars represent the standard deviation (SD). *p < 0.05 vs. Day 0, **p < 0.001 vs. Day 0.

To study the effects of cytokines in the thyroid microenvironment on IgG glycosylation, we first set up a two-step *in vitro* differentiation culture system. Peripheral blood mononuclear cells (PBMCs) from healthy donors were separated by density gradient centrifugation over Ficoll-Paque PLUS (GE Healthcare Life Sciences, USA). Then, CD19^+^ B lymphocytes were purified by positive selection using magnetic cell separation (CD19 MicroBeads, Human; Miltenyi Biotech, Germany). The purity of the CD20^+^CD19^+^ B cell isolates was above 98% according to flow cytometry (**Figure 2A**).

CD19^+^ B cells were cultured in X-VIVO 15 serum-free medium (Lonza, Switzerland) supplemented with 100 U/mL penicillin, 100 U/mL streptomycin (Gibco, USA), 5 μg/mL human holo-transferrin (Sigma-Aldrich, USA), and 5 μg/mL insulin (Sigma-Aldrich, USA). It is known that the differentiation of ASCs in response to T cell-dependent antigens requires costimulatory signals to activate B cell receptors, CD40 ligands, and cytokines. Thus, we used the three essential signals to mimic T cell-dependent B cell activation and transformation. CD19^+^ B cells were cultured at 1×10^6^ cells/mL in 12-well plates and activated with 5 µg/mL F(ab’)_2_ fragment goat anti-human IgM (Jackson ImmunoResearch Laboratories, USA), 200 ng/mL recombinant MEGACD40L protein (soluble, human) (ENZO Life Sciences, USA), 2.5 μg/mL CpG oligodeoxynucleotide 2006 (*In vivo*Gen, USA), and 100 U/mL recombinant IL-2 and then incubated at 37 °C in a humidified atmosphere with 5% CO_2_ for 3 days. After 3 days of stimulation, the activated B cells were seeded into 96-well plates at 5×10^5^ cells/mL and cultured with 50 U/mL IL-2, 50 ng/mL IL-10, and 20 ng/mL IL-4 as the control culture conditions. The differentiation efficiency of ASCs was confirmed by flow cytometry.

B cells were washed and resuspended in phosphate-buffered saline (PBS) and incubated with 7-aminoactinomycin D (7-AAD) for 20 min at room temperature (RT) to exclude dead cells. Single-cell suspensions were then washed and stained with optimal dilutions of FITC-CD19, PE-CD20, PerCP-CD38, BV510-CD27 (BD, USA), and APC-CD138 in the dark for 30 min at room temperature (RT). Isotype-matched antibodies were used in all procedures as negative controls. The above antibodies were obtained from Biolegend, Inc., unless otherwise indicated. Then, the cells were washed for further flow cytometry analysis with a FACSCanto II flow cytometer and FlowJo software (Version 10, FlowJo, USA).

### Stimulation of B Cells *In Vitro*

The sorted B cells were cultured to differentiate into ASCs under control culture conditions, and IFN-γ, TNF-α, IL-21, IL-17A, BAFF, and APRIL (Peprotech, USA) were individually added to the control culture system in a concentration gradient during the differentiation period. The culture supernatants at day 12 were collected and stored at -80°C. At the same time, the cells were harvested, washed, and stored at -80°C for further experiments. Experiments involving stimulation with each cytokine were performed at least three times with different donors in three replicates.

### Measurement of IgG in Supernatants by ELISA

The concentrations of total IgG in the supernatants were measured by enzyme-linked immunoassay (ELISA). Briefly, anti-human IgG (1:400 dilution; Abcam, UK) antibodies were precoated onto a plain 96-well plate at 4°C overnight. Then, the culture samples and IgG standards (Sigma-Aldrich, USA) were incubated with capture antibodies after blocking with 3% bovine serum albumin (BSA) at 37°C for 1 h. Diluted horseradish peroxidase (HRP)-conjugated detection antibody (1:2,000 dilution; Abcam, UK) was added to each well after washing to remove unbound protein, and the plate was maintained at 37°C for 1 h. After washing, hydrogen peroxide and o-phenylenediamine (OPD, Sigma-Aldrich, USA) in citrate–phosphate buffer (pH=5.0) were used as substrate solutions to react with HRP, and the absorbance at 492 nm was recorded with a microplate reader. The concentrations of IgG were determined according to the standard curve of serial dilutions.

### Detection of IgG Glycosylation by High-Density Lectin Microarrays

We used commercial lectin microarrays from BCBIO (Guangzhou, Guangdong, China) to detect the glycosylation of IgG in culture supernatants under different stimuli and controls. The details of the lectin microarrays are shown in **Table S1**. The specificities and sources of lectins used in the microarray have been reported in a previous study (37). The microarray was blocked with 50 mM ethanolamine in borate buffer (pH=8.0), and then the IgG concentrations of all samples were adjusted to 5.1 μg/mL with PBST buffer (PBS buffer with 0.05% TWEEN-20) for microarray detection. Alexa Fluor 647-conjugated goat anti-human IgG (H+L) cross-adsorbed antibody (Invitrogen, CA, USA) was oxidized by 20 mM sodium periodate and hybridized with the microarray for 1 h at RT. A Lux Scan 10K-A scanner (CapitalBio Corporation, China) was used to scan the microarray at 10-μm resolution with preadjusted parameters as follows: a 700-photomultiplier tube (PMT) and a power of 75 for the Cy5 channel. The lectin microarray images were converted to numerical format using LuxScan 3.0 software.

The signal-to-noise ratio (S/N) of each lectin spot was obtained for further screening and statistical analysis. Each lectin on the microarray was present in triplicate, and the signal intensities from replicate lectin measurements in which the coefficient of variation was less than 30% were accepted and averaged. For the negative control in each microarray, the 95% confidence interval of the S/N was 0.8-1.2. Consequently, detectable signals were retained only if the S/N was greater than or equal to 1.2, and any undetected signal was set as 1.0 (37).

### Purification of IgG From Culture Supernatants

Culture supernatants were filtered through a 0.22 μm filter (Millipore, USA), and then Hitrap Protein G HP (1 mL, GE Healthcare, USA) was used to purify IgG according to a previously published procedure (9). Briefly, culture samples stimulated with various cytokines were pumped into the affinity column, and unbound proteins were removed using five column volumes of binding buffer (0.02 MTris, pH=7.2). Bound IgG was eluted with elution buffer (0.1 M glycine, pH=2.7), and the eluate was neutralized to pH 7.2 immediately. Then, IgG samples were desalted and exchanged in PBS buffer using a PD-10 Desalting Column (GE Healthcare, USA) according to the manufacturer’s instructions, and the IgG solutions were concentrated using a 10-kD ultrafiltration tube (Millipore, USA). The purified IgG samples were stored at -80°C until further use.

### N-Glycosylation Profile Analysis of Purified IgG Using MALDI-TOF-MS

The N-glycans of purified IgG in the supernatants were released by PNGase F glycosidase (New England Biolabs, MA) according to our previous protocol (9). Briefly, 40 μg purified IgG sample was added to glycoprotein denaturing buffer (5% SDS, 0.4 M dithiothreitol) and heated at 100°C for 10 minutes. Then, the denatured sample was added to 10% Nonidet P 40 (NP-40) and incubated with 5 μL PNGase F at 37°C for 3 h. The released glycans of IgG were purified by UniElut graphitized carbon solid phase extraction (SPE) columns (Acchrom Technologies Co., Ltd., China). The sample was then lyophilized for the following procedure.

The glycan samples were permethylated and purified according to a previously reported study (38). Briefly, lyophilized samples were mixed with sodium hydroxide slurry in dimethyl sulfoxide and methyl iodide and vortexed for 20 min at RT. After adding chloroform and deionized water (DI water, 18.2 MΩ·cm), the samples were mixed thoroughly and centrifuged. Afterward, the upper aqueous layer was removed, and the chloroform layer was lyophilized. Permethylated glycans were dissolved in 50% methanol, purified on a C18 Sep-Pak 96-well cartridge (Waters, Milford, MA) and lyophilized again. Afterward, the derivatized glycans were dissolved in 10 μL methanol. One microliter of a matrix solution containing 10 mg/mL 2,5-dihydroxybenzoic acid (DHB) was mixed with 1 μL of derivatized glycan, added to a μfocus matrix-assisted laser desorption/ionization (MALDI) plate target (900 μm, 384 circles, HST), and then air-dried at RT. MALDI-mass spectrometry (MALDI-MS) data were obtained in positive mode with a power setting of 130, a mass range from m/z 1000 to 4000U, and 250 shots per sample using a MALDI-time of flight (MALDI-TOF) mass spectrometer (Shimadzu Axima Resonance). All detected glycan peaks are listed in **Table 1**. The frequency of derived glycosylation traits was calculated as described in **Table S2** according to the previous studies (39, 40), including fucosylated N-glycans (F), agalactosylated N-glycans (G0), mono-galactosylated N-glycans (G1), digalactosylated N-glycans (G2), asialylated N-glycans (S0), mono-sialylated N-glycans (S1), disialylated N-glycans (S2), and bisecting GlcNAc (B).

**Table 1:** The fine structures of 12 IgG N-glycans detected by MALDI-QIT-TOF-MS.

No.	Caculated [M+Na]^+^ m/z^1^	Detected [M+Na]^+^ m/z	Structure Abbreviation	Glycan Structure^2^	Relative Indensity (%)						
					Control	IFN-γ	TNF-α	IL-21	IL-17A	BAFF	APRIL
1	1835.9	1835.69	G0F	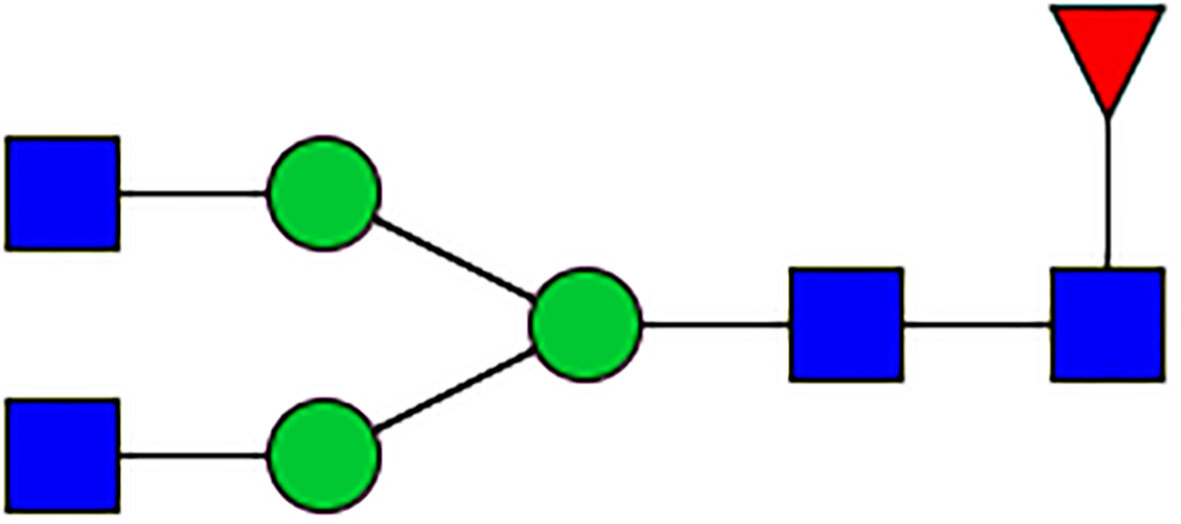	18.1	15.4	13.5	15.1	15.5	16.9	12.7
2	2040.0	2039.75	G1F	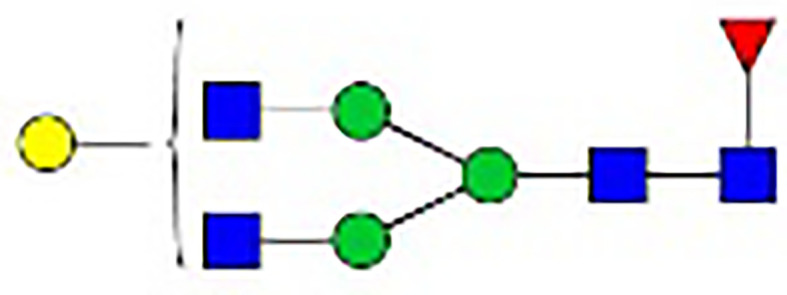	40.8	35.9	25.7	26.8	31.5	41.2	50.4
3	2070.0	2069.75	G2	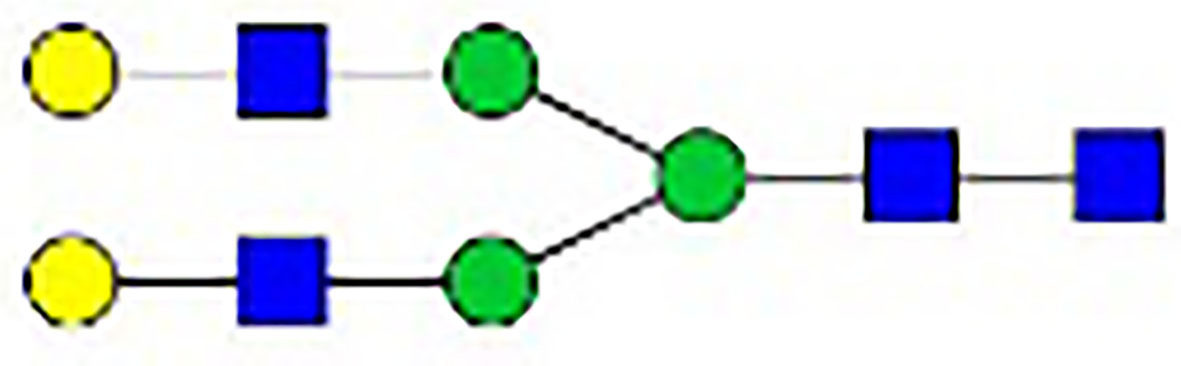	-^3^	0.4	0.4	0.2	0.5	–	–
4	2081.1	2080.8	G0FN	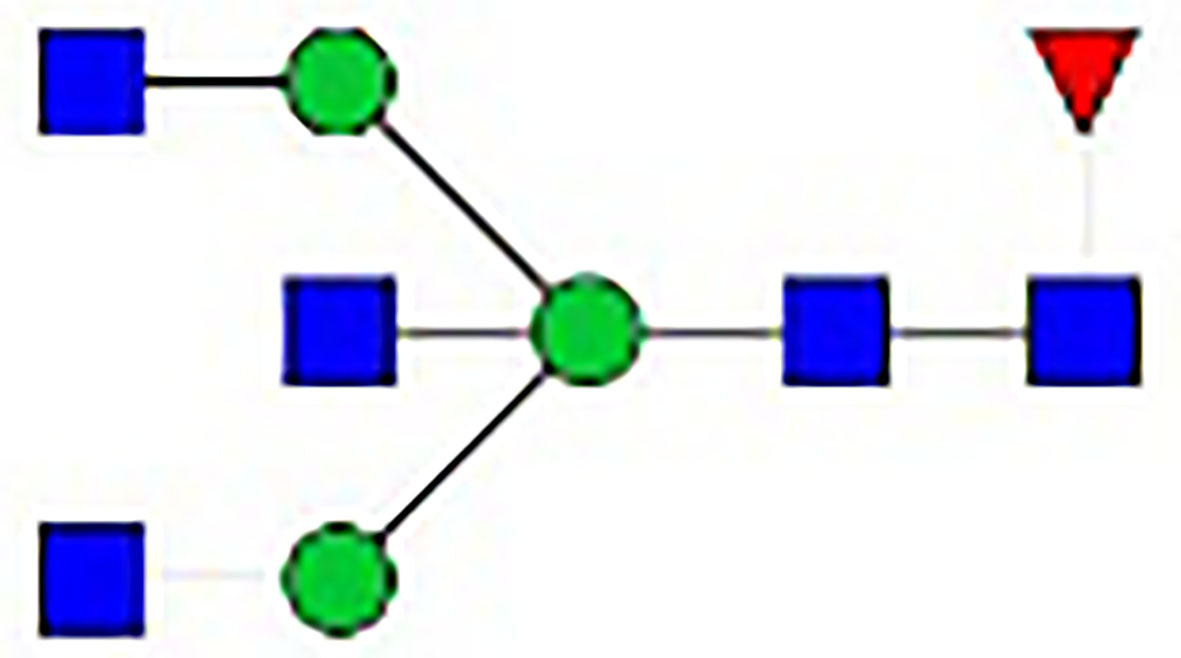	6.3	3.8	6.5	4.9	3.6	6.6	3.4
5	2244.1	2243.79	G2F	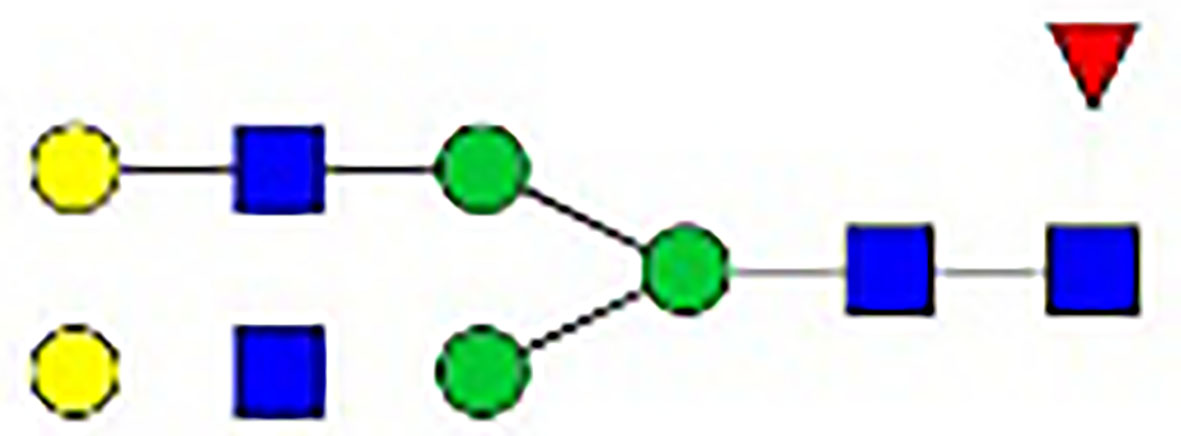	24.6	8.2	6.1	6.4	6.4	12	10.6
6	2285.2	2284.88	G1FN	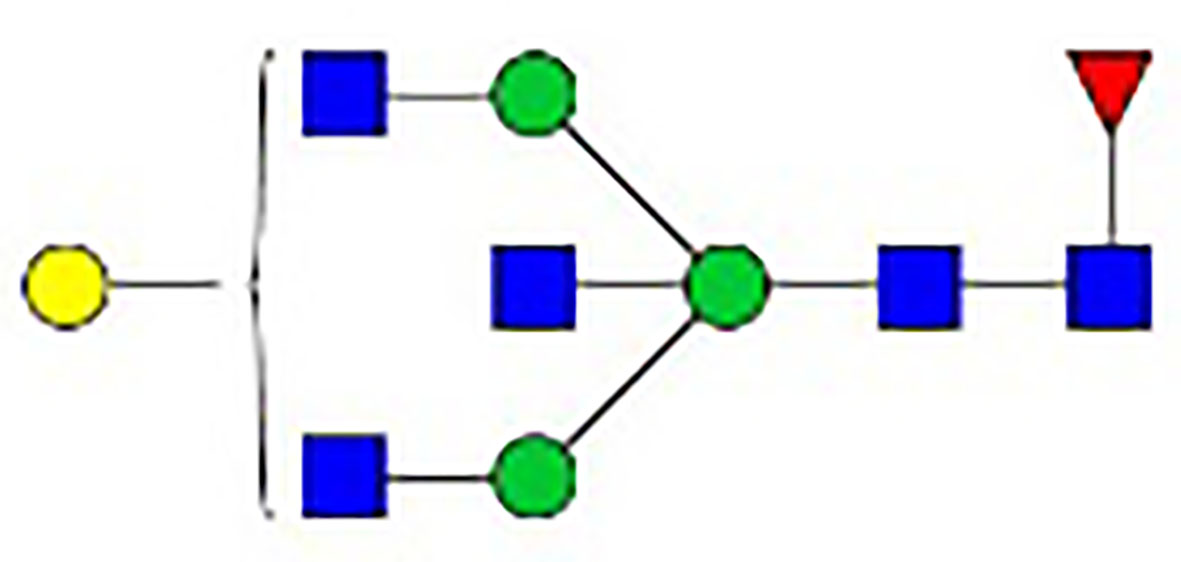	5.9	35	43	39	35.8	21.2	21.9
7	2401.2	2400.86	G1FS1	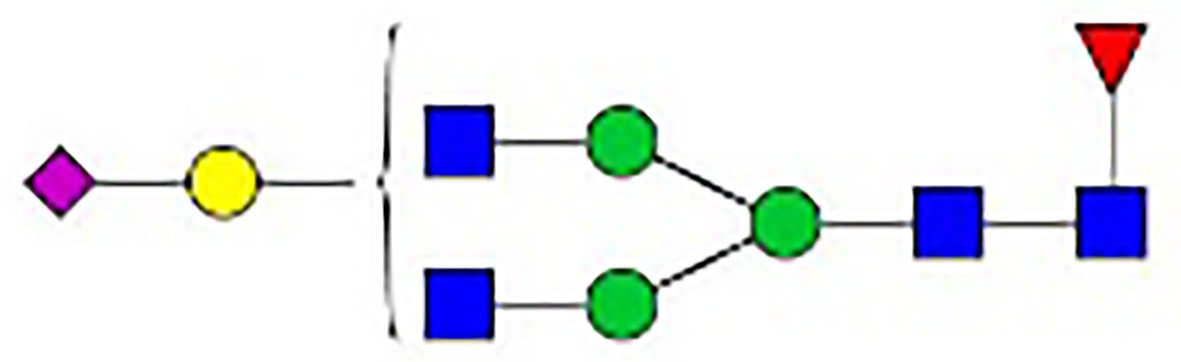	–	–	0.7	0.9	0.6	–	–
8	2489.3	2488.93	G2FN	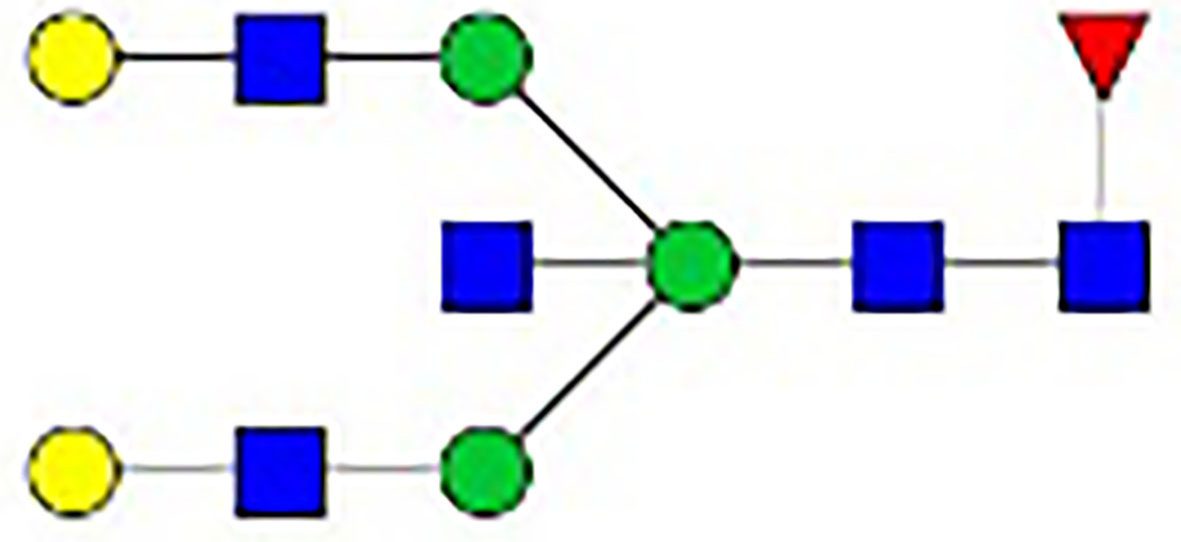	–	0.8	0.5	0.2	–	0.9	0.6
9	2605.3	2604.88	G2FS1	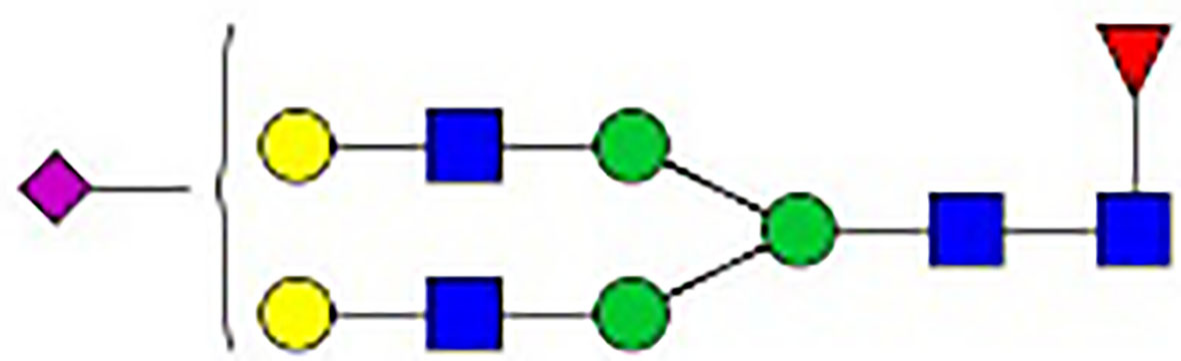	4.2	0.3	2.9	5.5	4.4	0.8	0.3
1-	2646.3	2645.96	G1FNS1	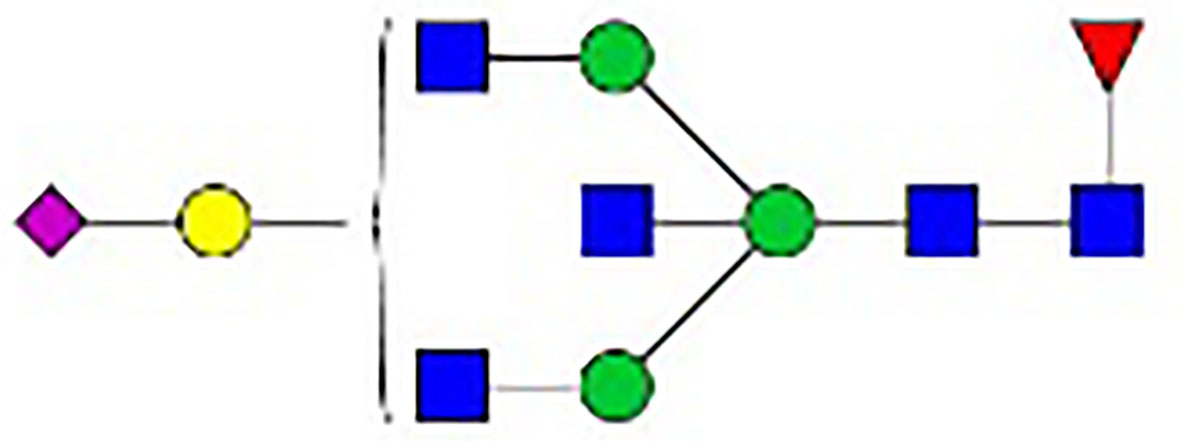	–	–	–	0.2	0.1	–	–
11	2850.4	2850.05	G2FNS1	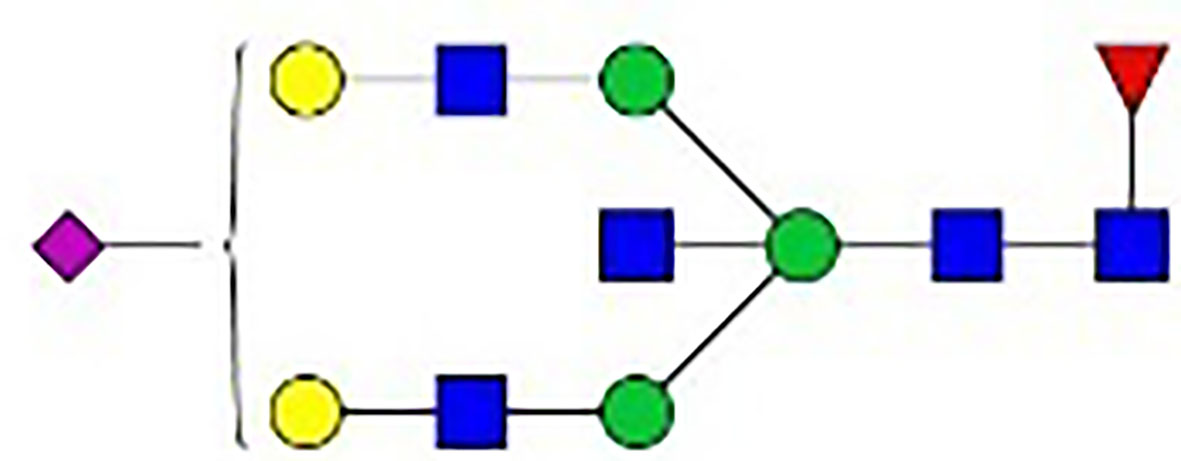	–	–	0.4	0.4	0.5	0.2	–
12	3211.6	3211.11	G2FNS2	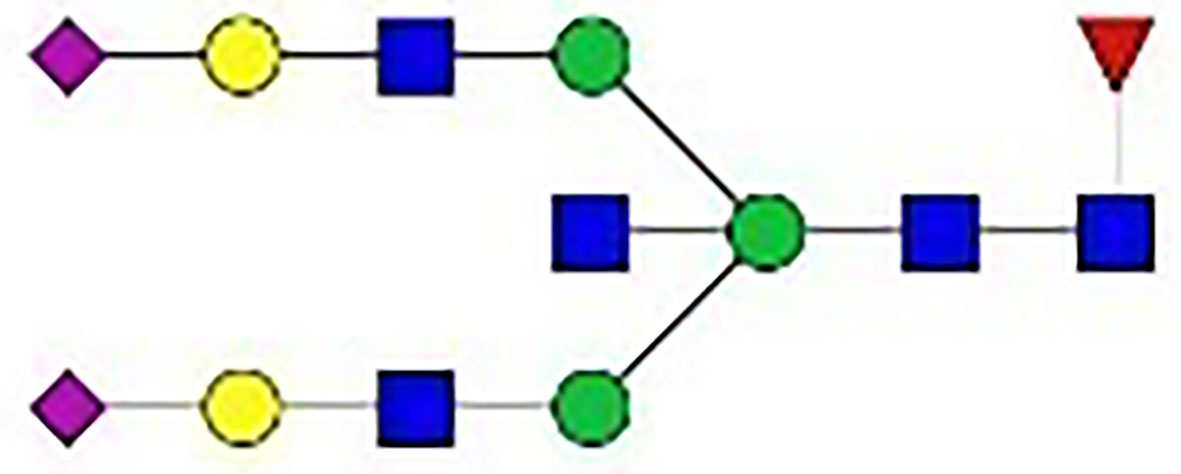	–	–	–	0.4	1	–	–

^1^Permethylated mass value;

^2^Glycan structural features: blue squares, N-acetylglucosamine (N); red triangles, fucose (F); green circles, mannose (M); yellow circles, galactose (G); purple rhombus, sialic acid (S).

^3^The undetected N-glycan signals were represented as “-”.

### Identification of Glycosylation in F(ab’)_2_ and Fc Fragments by Lectin Blotting Under Control Culture Conditions

First, the purified IgG under control conditions was adjusted to a concentration of 1 μg/μL with digestion buffer (50 mM sodium phosphate, 150 mM NaCl, pH=6.6). Immunoglobulin-degrading enzyme from *Streptococcus pyogenes* (IdeS protease, Promega, USA) was added to the IgG samples at a ratio of 1:1 (1 μg IgG/1 unit IdeS Protease), and then the samples were incubated at 37°C for 60 minutes. The mixture of F(ab’)_2_ and Fc fragments from IgG was immediately placed on ice. Then, the digestion efficiency of IdeS protease was determined by nonreducing sodium dodecyl sulfate–polyacrylamide gel electrophoresis (SDS-PAGE). Samples of F(ab’)_2_ and Fc fragments were diluted to a concentration of 0.5 μg/μL in nonreducing loading buffer (Beyotime, China) containing 5% SDS. Furthermore, the samples were denatured by heating at 70°C for 5 min. A total of 2 μg IgG sample per well was loaded for nonreducing SDS-PAGE with 4-12% ExpressPlus™-PAGE gels (GenScript, USA). The gels were washed with deionized water three times, stained with BeyoBlue™ Coomassie Blue Super Fast Staining Solution (Beyotime, China) and photographed with GBOX-Chemi XT4 (Syngene, UK).

We used *Sambucus nigra lectin* (SNA) to identify glycosylated fragments, since both IgG Fab and Fc fragments contain various percentages of sialylation (4). Electrophoresis was performed on 4-12% ExpressPlus™-PAGE gels (GenScript, USA) to separate the F(ab’)_2_ and Fc fragments, and then the samples were transferred to BioTrace™ NT nitrocellulose (NC) membranes (Pall Corporation, USA), which were blocked with 3% BSA at 4°C overnight. Biotinylated SNA lectin (1:1,000 dilution, Vector Laboratories, USA) was incubated with the NC membranes at RT for 2 h. Streptavidin peroxidase was diluted 1:2,000 in 3% BSA for 1 h at RT. SuperSignal West Femto maximum-sensitivity substrate (Thermo Scientific, USA) was used to visualize the bands, and the membrane was scanned by GBOX-Chemi XT4 (Syngene, UK).

### Measurement of Glycosylation Enzymes by Quantitative Real-Time PCR

Cytokines that significantly altered the glycosylation of IgG were selected for coculture with B cells, and then the mRNA expression of glycosylation enzymes in B cells was detected by real-time PCR. Total RNA was extracted from stimulated B cells by TRIzol reagent (Life Technologies, USA) and reverse-transcribed into cDNA using the High-Capacity cDNA Reverse Transcription Kit (Applied Biosystems, USA). Quantitative real-time PCR was carried out with a 7500 Fast Real-Time PCR System using SYBR Green Master Mix (Applied Biosystems, USA). The optimal primer sequences were obtained from the Primer-Blast tool from NCBI (National Center for Biotechnology Information, National Institutes of Health, http://www.ncbi.nlm.nih.gov/tools/primer-blast/) and Primer Bank (http://pga.mgh.harvard.edu/primerbank/), as listed in **Table 2**. Experiments involving stimulation with each cytokine and control conditions were performed with at least three batches from different donors in three replicates.

**Table 2:** Primer sequences for the glycosylation enzymes.

Gene name	Forward Primer	Reverse Primer
ST6GAL1	5′- AACTCTCAGTTGGTTACCACAGA-3′	5′- GGTGCAGCTTACGATAAGTCTT-3′
NEU1	5′- CTTTGCTGAGGCGAGGAAAAT-3′	5′- TTGACAATGAACGCTGTAGGAG-3′
B4GALT1	5′- CCAGGCGGGAGACACTATATT-3′	5′- CACCTGTACGCATTATGGTCAT-3′
GLB1	5′- GTGCTGTACCGGACAACACTT-3′	5′- ATCACATTGTTTCGCTCAAGGA-3′
GAPDH	5′-GGAGCGAGATCCCTCCAAAAT-3′	5′- GGCTGTTGTCATACTTCTCATGG-3′

Sequences of the glycosylation enzyme primers used in the study for real-time quantitative PCR detection.

### Measurement of Glycosylation Enzymes by Western Blotting

B cells were harvested and lysed on ice for 15 min in RIPA lysis buffer (Pulilai Gene Technology, Beijing) supplemented with a protease inhibitor cocktail (MedChemExpress, USA). Cell lysates (20 µg per sample) were denatured and subjected to SDS-PAGE (10%), followed by transfer to polyvinylidene fluoride (PVDF) membranes. The membranes were blocked with 5% skim milk and subsequently immunoblotted with anti-B4GALT1 antibody (1:250 dilution; Abcam), anti-GLB1 antibody (1:1000 dilution; Abcam), anti-CD75/ST6GAL1 antibody (1:1,000 dilution; Abcam), anti-sialidase-1 (NEU1) antibody (1:2,000 dilution; Abcam), and anti-GAPDH antibody (1:2,000 dilution, TransGen Biotech, China) overnight at 4°C. HRP-conjugated secondary antibodies were added for incubation, and then blots were developed with SuperSignal West Femto Maximum Sensitivity Substrate (Thermo Scientific, USA). ImageJ software (developed at the National Institutes of Health) was used to quantify the relative staining intensity. Each experiment was conducted with at least three batches from different donors.

### Statistical Analysis

All analyses were performed using Prism 8 software (GraphPad, USA). All experiments were performed at least three times from different donors with three replicates. Statistical analysis was performed with an unpaired Student’s t-test or one-way analysis of variance (ANOVA), as appropriate. A *p* value less than 0.05 was considered statistically significant.

## Results

### Differentiation of B Lymphocytes Into ASCs *In Vitro*

Unlike previous studies on PBMCs, we cultured human primary B cells and used a two-step *in vitro* differentiation culture system to simulate the microenvironment of AIDs (**Figure 1C**). In this system, we found that up to 38.5% and 39.1% of CD19^+^ B cells had differentiated into plasma cells and plasmablasts, respectively, at day 12 (**Figures 2B, C**). We determined day 12 as the harvest day (**Figure 2D**) under optimized culture conditions (**Figures S1A–G**), which were applied as the control culture conditions in the subsequent experiments.

### Production of IgG in Culture Supernatants Under Different Cytokines

**Figure 3:**
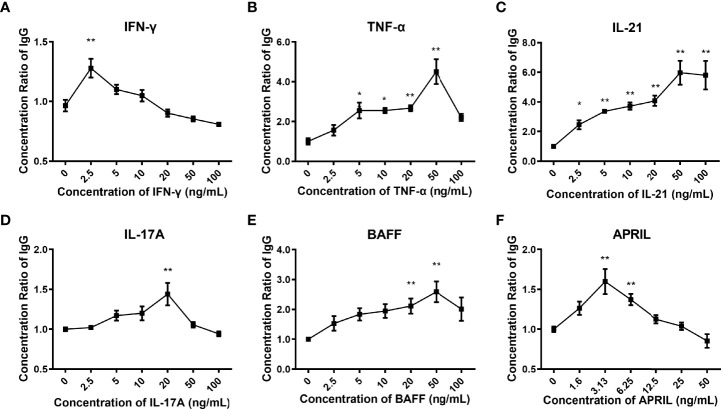
Production of IgG in the culture supernatants. **(A–F)** Concentration ratios of IgG in the culture supernatants stimulated by different concentrations of IFN-γ, TNF-α, IL-21, IL-17A, BAFF, and APRIL. The concentration ratio of IgG was calculated as the concentration of IgG under the specific stimulation divided by the basic concentration of IgG under the control condition. The error bars represent the SEM. *p < 0.05 vs. 0 ng/ml group, **p < 0.001 vs. 0 ng/ml group.

We found that the addition of IFN-γ, TNF-α, IL-21, IL-17A, BAFF, and APRIL to the control culture medium could promote the secretion of IgG (*p*<0.001). IL-21 increased the yield of IgG in a concentration-dependent manner (**Figure 3C**). The production of IgG in response to the other cytokines varied according to the concentration, presenting an inverted U-shaped curve (**Figures 3A, B, D–F**). The following cytokine concentrations were selected for subsequent experiments according to the peak yield of IgG: 2.5 ng/mL for IFN-g, 50 ng/mL for TNF-a, 50 ng/mL for IL-21, 20 ng/mL for IL-17A, 50 ng/mL for BAFF, and 3.13 ng/mL for APRIL.

### Glycosylation Patterns of IgG Detected by Lectin Microarray

**Figure 4:**
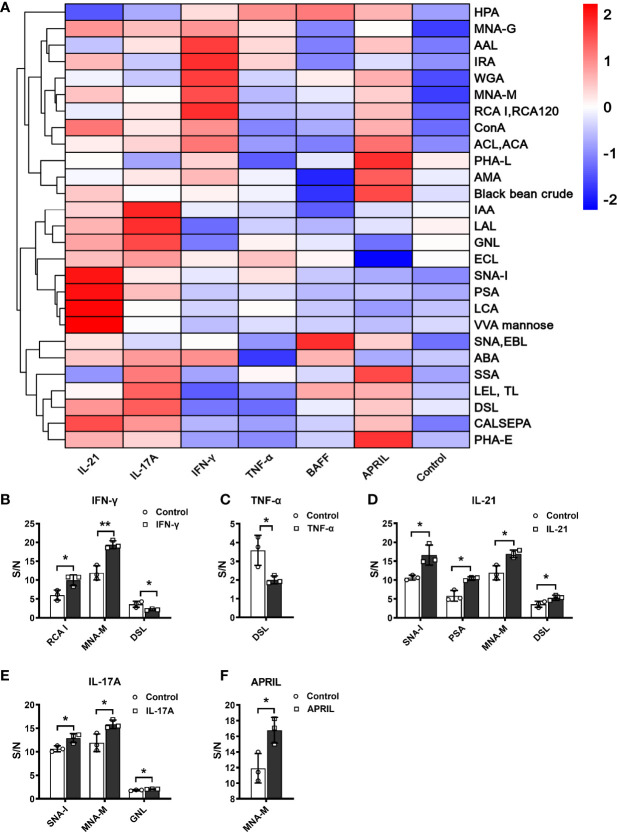
S/N of detectable lectins for IgG under different cytokine stimulations. **(A)** Clustered heatmap of lectin IgG binding profiles. Detectable lectins were defined as those with (1) an S/N > 1.2 for each lectin spot and (2) an S/N coefficient of variation (measured in triplicate) less than 30%. The detectable lectins are listed on the left vertical axis, and the samples under different stimuli are indicated along the horizontal axis, including IFN-γ, TNF-α, IL-21, IL-17A, BAFF, and APRIL. Each square represents the S/N of a lectin binding pattern on IgG. The values of S/N were transformed by min–max normalization. The color bar represents the scale—red indicates a higher S/N, and blue indicates a lower S/N. **(B–F)** S/N of IgG with significant changes under stimulation by IFN-γ, TNF-α, IL-21, IL-17A, and APRIL *in vitro*. The binding specificities of lectins were SNA-I, SNA, and EBL for terminal Neu5Acα2–6/sialic acid (SA); RCA-I, ECL, PHA-L, PHA-E, MNA-G, ACL, ACA, and ABA for galactose (Gal); and DSL, LEL, TL, and WGA for N-acetylglucosamine (GlcNAc). The error bars represent the SD. *p < 0.05, **p < 0.001.

As shown in **Figure 4A**, the glycosylation patterns of IgG in the supernatants showed considerable differences under stimulation with different cytokines. The recognition specificities and sources of the lectins are listed in **Table 3**. Each cytokine played a unique role in regulating the glycosylation profile of IgG. Specifically, IFN-γ upregulated the levels of galactose (RCA-I *p*<0.05) and mannose (MNA-M *p*<0.05) and decreased the level of GlcNAc (DSL, *p*<0.05) (**Figure 4B**). TNF-α decreased the level of GlcNAc (DSL, all *p*<0.05) (**Figure 4C**). IL-21 significantly increased the levels of sialic acid (SNA-I *p*<0.05), mannose (PSA *p*<0.05, MNA-M *p*<0.05), and GlcNAc (DSL *p*<0.05) (**Figure 4D**). The addition of IL-17A increased the levels of sialic acid (SNA-I *p*<0.05) and mannose (MNA-M, GNL all *p*<0.05) (**Figure 4E**). APRIL significantly increased the mannosylation of IgG (MNA-M, *p*<0.05) (**Figure 4F**). The addition of BAFF showed no influence on IgG glycosylation. In short, the level of galactose was upregulated by IFN-γ; sialylation of IgG was upregulated by IL-21 and IL-17A; mannosylation of IgG was upregulated by IFN-γ, IL-21, IL-17A, and APRIL; and the level of GlcNAc was regulated by IFN-γ, TNF-α, and IL-21. All changes of glycosylation on IgG stimulated by various cytokines in lectin microarray are listed in **Table S3**.

**Table 3:** The recognition specificity and source of lectin.

Glycan	Full name	Abbr.	Preferred sugar	Source
Sialic acid	*Sambucus nigra* (elderberry bark)	SNA-I	α-2,6 linked sialic acid residues	4
	*Sambucus nigra lectin*	SNA, EBL	Neu5Acα6Gal/GalNAc	2
	*Salvia sclarea*	SSA	NeuAc	4
Galactose	*Morniga G lectin* (black elderberry)	MNA-G	Gal	4
	*Ricinus communis agglutinin I*	RCA I	Gal	4
	*Phaseolus vulgaris leucoagglutinin*	PHA-L	Galβ4GlcNAcβ6 (GlcNAcβ2 Manα3) Manα3	1
	*Phaseolus vulgaris Erythroagglutinin*	PHA-E	Galβ4GlcNAcβ2 (Galβ4GlcNAcβ6) Man	4
	*Erythrina cristagalli Lectin*	ECL	Galβ4GlcNAc (Terminal)	4
	*Agaricus bisporus Lectin* (Mushroom)	ABA	Galβ3GalNAc	4
	*Amaranthus caudatus lectin*	ACL,ACA	Galβ3GalNAc	4
Fucose	*Aleuria aurantia lectin*	AAL	Fucα6GlcNAc	2
	*Laburnum anagyroides lectin* (gold chain)	LAL	α-Me-L-Fucose among monosaccharides	4
	*Lens Culinaris Agglutinin*	LCA	Fucose linked α(1,6) to core GlcNAc of N-linked glycopeptides	3
Mannose	*Pisum sativum agglutinin*	PSA	αMan,αGlc	4
	*Morniga M lectin* (black elderberry)	MNA-M	Man	4
	*Galanthus nivalis* (snowdrop) *lectin*	GNL	αMan	2
	*Canavalia ensiformis, jack bean*	ConA	αMan,αGlc	4
	*Vicia villosa Lectin* (Hairy Vetch, Mannose Specific)	VVA Man	Man	4
	*Calystega sepiem Lectin* (Hedge Bindweed Rhizomes)	CALSEPA	Man	4
GlcNAc	*Datura stramonium lectin*	DSL	(GlcNAc)2-4	3
	*Triticum vulgare lectin* (wheat germ)	WGA	GlcNAcβ4GlcNAc)1-4	1
	*Lycopersicon esculentum lectin*	LEL,TL	GlcNAc (prefer trimer and tetramer)	3
GalNAc	*Helix pomation Lectin* (Snail)	HPA	GalNAc	1
	*lris hybrid Lectin* (Dutch Iris)	IRA	GalNAc	4
	*Phaseolus vulgaris* sp. *Lectin*	Black bean crude	GalNAc> galactose, sialic acid	4
	*Iberis amara Lectin*	IAA	GalNAc	4

Detailed information on the detectable lectins is listed above.

The lectins used in this study were obtained from four different sources: 1, Molecular Probes, Inc.; 2, Irwin J. Goldstein’s group; 3, Vector Laboratories, Inc.; and 4, EY Laboratories, Inc. Sugar abbreviations: Fuc, fucose; Gal, galactose; GalNAc, N-acetylgalactosamine; Glc, glucose; GlcNAc, N-acetylglucosamine; Man, mannose; Neu5Ac, N-acetylneuraminic acid (sialic acid).

### N-Glycan Structure Analysis by MALDI-TOF-MS

**Figure 5:**
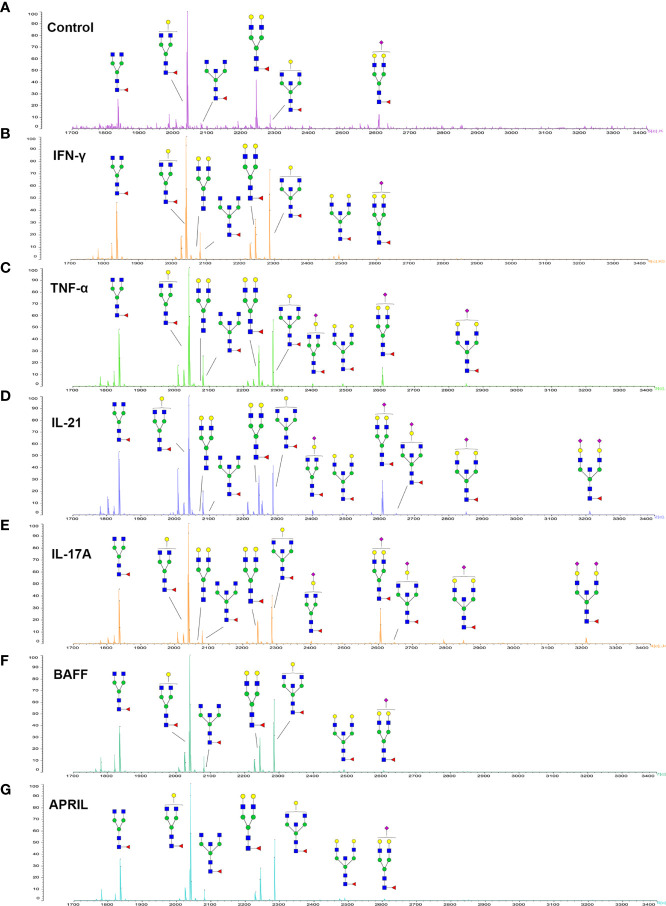
MALDI-TOF-MS spectrum of N-glycans from purified IgG stimulated by cytokines. **(A–G)** Glycosylation profiles of purified IgG from the culture medium under control conditions and stimulation with IFN-γ, TNF-α, IL-21, IL-17A, BAFF, and APRIL detected by MALDI-TOF-MS at m/z 1700–3400.

Since galactose, sialic acid, mannose, and GlcNAc were attached to the heptasaccharide core structure in various manners, the fine structure of various glycoforms could not be distinguished by lectin microarray. MALDI-TOF-MS was further performed to confirm the carbohydrate structures of glycans released from IgG. A total of 12 distinct N-glycan structures were identified and annotated with the proposed structures shown in **Table 1**. The relative intensities of derived glycosylation traits are listed in **Table 4**. Though the increased level of mannose was detected by lectin microarray, we found that all detectable glycoforms were complex-type N-glycans (**Figure 5**). This could be associated with the sample preparation causing a great loss, the amounts of terminal mannose-containing N-glycoform was too low to be detected by MALDI-TOF-MS. An overlap of 6 N-glycan peaks was shown in the control and stimulation groups (G0F m/z 1835.69, G1F m/z 2039.75, G0FN m/z 2080.8, G2F m/z 2243.79, G1FN m/z 2284.88, and G2FS1 m/z 2604.88).

**Table 4:** Relative intensity of derived glycosylation traits.

Derived traits	Relative Intensity (%)						
	Control	IFN-γ	TNF-α	IL-21	IL-17A	BAFF	APRIL
**Fucosylated N-glycans (F)**	99.9	99.4	99.3	99.8	99.4	99.8	99.9
**Agalactosylated N-glycans (G0)**	24.4	19.2	20.0	20.0	19.1	23.5	16.1
**Mono-galactosylated N-glycans (G1)**	46.7	70.9	69.4	66.9	68	62.4	72.3
**Digalactosylated N-glycans (G2)**	28.8	9.7	10.3	13.1	12.8	13.9	11.5
**Asialylated N-glycans (S0)**	95.7	99.5	95.7	92.6	93.3	98.8	99.6
**Mono-sialylated N-glycans (S1)**	4.2	0.3	4.0	7.0	5.6	1.0	0.3
**Disialylated N-glycans (S2)**	0	0	0	0.4	1.0	0	0
**Bisecting GlcNAc (B)**	12.2	39.6	50.4	45.1	41.0	28.9	25.9

More detectable N-glycoforms containing galactose were observed in IFN-γ group than in control group, including G1F (m/z 2039.75), G2 (m/z 2069.75), G2F (m/z 2243.79), G1FN (m/z 2284.88), G2FN (m/z 2488.93), and G2FS1 (m/z 2604.88) (**Figure 5B**). We found a decreased frequency of G0 in the IFN-γ group (19.2%) compared to the control group (24.4%), and a higher frequency of G1 in the IFN-γ group (70.9%) than in the control condition (46.7%). This result indicated that the increased frequency of G1 may contribute to the increased binding of IgG to RCA-I, which recognized galactose in lectin microarray.

The addition of IL-21 and IL-17A enriched sialylated N-glycan structures (including G1FS1 m/z 2400.86, G2FS1 m/z 2645.96, G2FNS1 m/z 2850.05, and G2FNS2 m/z 3211.11) (**Figures 5D, E**). We found that the frequency of S0 was decreased by IL-21 and IL-17A compared to the control condition (92.6% in the IL-21 group; 93.3% in the IL-17A group; 95.7% in the control group). And the frequencies of both S1 (7.0% in the IL-21 group; 5.6% in the IL-17A group; 4.2% in the control group) and S2 (0.4% in the IL-21 group; 1.0% in the IL-17A group; undetected in the control group) were higher in IL-21 and IL-17A groups, which was consistent with the data in lectin microarray.

We further identified bisecting GlcNAc glycoforms by MALDI-TOF-MS, including G0FN (m/z 2080.8), G1FN (m/z 2284.88), G2FN (m/z 2488.93), G1FNS1 (m/z 2850.05), and G2FNS2 (m/z 3211.11). We found that the relative intensity of bisection glycoforms was higher in the stimulation groups (39.6% in the IFN-γ group; 50.4% in the TNF-α group; 45.1% in the IL-21 group; 41.0% in the IL-17A group; 28.9% in the BAFF group; 25.9% in the APRIL group) than in the control group (12.2%).

Combined with the results of fine structural analysis by MALDI-TOF-MS and the quantification of lectin microarray, we finally confirmed that the proportion of galactose-containing glycans, especially G1, was increased by IFN-γ and that the frequency of sialylated glycoforms was increased by IL-21 and IL-17A.

### Regulation of Glycosylation Enzymes by Different Cytokines

We further explored changes in the mRNA and protein levels of galactose- and sialic acid-related glycosyltransferases and glycosylhydrolases under stimulation with IFN-γ, IL-21, and IL-17A. The main functions and details of the glycosylation enzymes are shown in **Table S4**.

**Figure 6:**
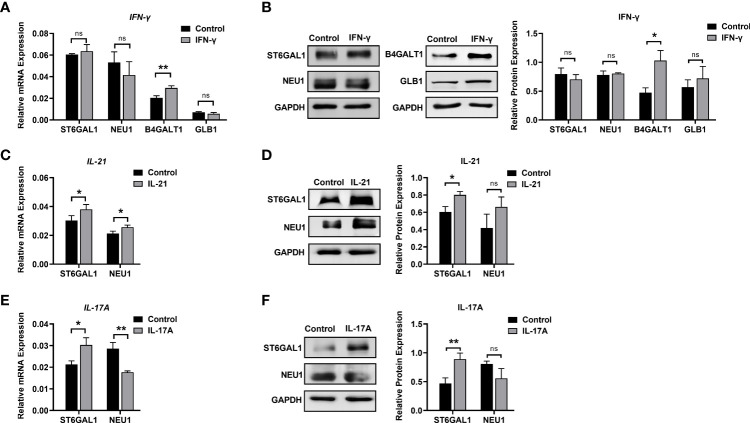
Changes in glycosylation enzymes in B cells under stimulation. **(A)** mRNA expression of B4GALT1, GLB1, ST6GAL1, and NEU1 under stimulation with IFN-γ. **(B)** Protein expression of B4GALT1, GLB1, ST6GAL1, and NEU1 under stimulation by IFN-γ. **(C, E)** mRNA expression of ST6GAL1 and NEU1 under stimulation by IL-21 and IL-17A, respectively. **(D, F)** Protein expression of ST6GAL1 and NEU1 under stimulation by IL-21 and IL-17A, respectively. Total RNA was extracted from activated B cells after 12 h of stimulation. Protein was also collected from activated B cells after 72 h of stimulation. The mRNA and protein expression of glycosylation enzymes was normalized to the GAPDH mRNA and GAPDH protein relative staining intensity. The error bars represent the SD. ns means no significance, *p < 0.05, **p < 0.001.

Elevated of IgG galactosylation was observed when B cells were stimulated with IFN-γ. Both the mRNA (1.45-fold) and protein levels of β-1,4-galactosyltransferase 1 (B4GALT1, 2.18-fold) were increased in the IFN-γ group compared with the control group, while no changes were found in either the mRNA or protein levels of β-galactosidase (GLB1) between the IFN-γ and control groups (**Figures 6A, B**). As shown in **Figure 1B**, the absence of sialic acid may result in the exposure of galactose residues. We found no significant changes in the expression of ST6GAL1 and NEU1 in B cells after stimulation with IFN-γ (**Figures 6A, B**) at the transcriptional and translational levels. This result indicated that the increased galactosylation regulated by IFN-γ resulted from changes in B4GALT1 rather than the expression of ST6GAL1 and NEU1.

For sialic acid, we found that IL-21 significantly upregulated the mRNA expression of β-galactoside α-2,6-sialyltransferase 1 (ST6GAL1) by 1.25-fold and sialidase-1 (NEU1) by 1.2-fold (**Figure 6C**). The protein expression of ST6GAL1 was significantly increased to 1.33-fold, and no significant difference in NEU1 protein expression was detected after the addition of IL-21. The addition of IL-17A resulted in upregulation of ST6GAL1 mRNA and protein expression by 1.42-fold and 1.92-fold, respectively, and a 1.6-fold downregulation of NEU1 mRNA expression (**Figure 6E**) with no significant difference in NEU1 protein expression (**Figures 6D, F**). Our data showed that changes in galactose and sialic acid resulted from upregulation of glycosyltransferases rather than glycosylhydrolases.

## Discussion

Glycosylation, as a posttranslational modification of IgG, is critical for the modulation of the immune response to inflammation, including ADCC (41), CDC (42), antibody-dependent cellular phagocytosis (ADCP) (43), and antigen binding (4), and may act as a ‘switch’ to control the pathogenicity of IgG (15). In our study, we found that cytokines in the microenvironment promoted IgG secretion and changed the N-glycan patterns of IgG. Meanwhile, the expression of B4GALT1 and ST6GAL1 was upregulated to meet the demands for IgG glycosylation, including the increased amount of IgG and the level of sialylation and galactosylation. This study may provide a new perspective for the control of the IgG evolutionary direction to prevent the development of AIDs.

Accumulating evidence has identified a characteristic microenvironment with abnormally elevated cytokines and molecules in AID patients. In our research, the changes in glycosylation profiles of secreted IgG under stimulation with six cytokines during the differentiation phase were confirmed by lectin microarray and MALDI-TOF-MS. Data of lectin microarray showed that IFN-γ significantly increased the proportion of galactose-containing glycans, especially the G1 N-glycans detected by MALDI-TOF-MS. In addition, the upregulation of B4GALT1 after IFN-γ treatment further confirmed the changes of galactosylation of IgG. IL-21 and IL-17A significantly increased sialylation of IgG detected by lectin microarray and confirmed by mass spectrum, accompanied by upregulation of ST6GAL1. The discrepancy in GlcNAc levels between lectin microarray and MALDI-TOF-MS may result from the combined effect of bisecting and biantennary GlcNAc. MALDI-TOF-MS identified ab increased frequency of bisecting glycans in the stimulation groups compared to control group, which indicated that cytokines may regulate the synthesis of bisecting GlcNAc in IgG. The above results highlight that the intracellular glycosylation-regulating mechanisms of cytokines are complex and independent, forming a multiple regulatory network. In the literature, studies have indicated that the intracellular glycosylation regulatory effects of cytokines on different cell types (44–46) and species (17, 18) have discrepant results. In addition, the regulation of IgG glycosylation *in vivo* and *in vitro* differs since extrinsic glycosylation plays a critical role in the homeostasis of IgG (21, 47, 48). Changes in IgG glycosylation are dynamically but precisely regulated throughout the course of disease, and the combined influences of complex *in vivo* immunologic environments on human B cells and IgG glycans remain poorly defined and should be further studied.

Glycosylation may be introduced in variable domains of the IgG Fab fragment. Fab glycans showed a high percentage of sialic acid (up to 93%) compared to Fc N-glycans (49), since the spatial structure of the Fab fragment was more accessible to glycosyltransferases. To determine whether Fab glycosylation occurred under our two-step culture condition *in vitro*, SNA (which specifically recognizes sialic acid) lectin blotting was performed to determine the glycosylation of the F(ab’)_2_ fragment. We found that under control condition glycosylation in the F(ab’)_2_ portion was quite low compared to that in the Fc fragment. However, we found that the relative intensity of G2FNS2 glycoform, which was reported to be presented in Fab glycosylation (40, 50), was increased by IL-21 and IL-17A. And in previous study (17), the G2FNS2 glycoform was undetected in Fc glycan profile of IgG after IL-21 stimulation of B cells, which indicated that introduced glycans may exist in variable domain of IgG. In addition, the elevated mannosylation of IgG with unified concentration was also observed in IL-21 and IL-17A groups with no detection of high mannose or hybrid type by MALDI-TOF-MS, which indicated that the increased mannose levels of IgG may result from the introduced glycans in Fab fragment of IgG. Above findings indicated that cytokines may play a role in regulating introduced glycosylation in Fab fragment of IgG during the differentiation period of B cell response.

Upregulated levels of galactose and sialic acid in IgG Fc may affect the biological function of IgG (15). Changes in Fc glycoforms might be involved in disease onset as the trigger to control the final immune response of IgG. Several studies observed that terminal galactosylation increases the affinity to FcγRIIIa and complement component 1q (C1q), leading to enhancement of ADCC and CDC activity (41, 42). Sialylation of IgG has been reported to decrease its affinity to FcγRIIIa to reduce ADCC activity but enhance ADCP activity (15) and prolong the half-life of IgG in serum (51). In short, the regulatory effects of cytokines on IgG glycosylation are multifactorial and complex. According to the results in our study, some cytokines such as IFN-γ trigger the pathogenicity of IgG by remodeling the carbohydrate structure of IgG, while other cytokines, such as IL-21 and IL-17A, endow IgG with dual roles of pro- and anti-inflammatory glycan patterns. Cytokines in the microenvironment dynamically and accurately regulate the immune responses of IgG *in vivo*.

In contrast to extracellular IgG glycosylation, we focused on the effects of cytokines on IgG glycoforms and further confirmed that the increased levels of galactose and sialic acid in IgG were upregulated by B4GALT1 and ST6GALT1 in human B cells, which complemented the intracellular regulatory mechanisms of diverse IgG glycosylation. The regulatory effects of cytokines on glycosylation enzymes are examined in limited studies and are poorly understood and controversial. For example, IFN-γ increases sialylated N-glycan structures due to upregulated mRNA levels of sialyltransferases (ST6GAL1) in human bone marrow-derived mesenchymal stromal cells (MSCs) to influence migration and survival (52). However, another study reported that IFN-γ decreased the expression of ST6GAL1 in LPS-induced B cell cultures (44). In our study, we found that the addition of IFN-γ showed no significant regulatory effect on ST6GAL1. These discrepancies might result from the different activation modes under different disease contexts in various studies, which indicated that the expression of glycosyltransferases was regulated by a variety of factors and involved in controlling the biological functions of functional proteins in different cell models.

This study had several limitations. First, glycan peaks with low intensity were undetected in MALDI-TOF-MS, which may result from the limited amount of IgG secreted *in vitro*, and the intricate sample preparation process of IgG purification, desalting, and N-glycan release caused high sample loss. However, the obtained glycoforms have proven the complex effect of cytokines on IgG glycosylation profiles. Second, the limited differentiation efficiency of our culture conditions *in vitro* resulted in the obtained IgG being insufficient to perform the functional experiments. Another notable limitation was that our research only confirmed the multiple regulation of single cytokines on IgG glycosylation; thus, the integrated effects of cytokine combinations on IgG glycosylation should be detected in B cells from patients with AIDs in future.

In conclusion, we proved that cytokines in the microenvironment promoted the secretion of IgG and altered the carbohydrate structures of IgG by regulating the expression of intracellular glycosylation enzymes in B cells *in vitro*. Our study revealed the interconnected complexity of glycan regulatory networks in the microenvironment, which is an essential complement of the intracellular regulation mechanisms of IgG glycosylation.

## Data Availability Statement

The original datasets analyzed in the current study are available from the corresponding author on reasonable request.

## Ethics Statement

The studies involving human participants were reviewed and approved by Medical Ethics Committee of Peking University First Hospital. The patients/participants provided their written informed consent to participate in this study.

## Author Contributions

YC, Conceptualization, methodology, formal analysis, investigation, resources, writing - original draft, and project administration. ZG, methodology and formal analysis. ZS, methodology, formal analysis, and investigation. XZ and YH, investigation and resources. YGo, conceptualization and methodology. KZ, methodology and investigation. CQ, conceptualization and investigation. YL, conceptualization and formal analysis. YGa, conceptualization, project administration, writing - review and editing, and funding acquisition. JZ and XG, conceptualization, and supervision.

## Funding

This work was supported by the National Natural Science Foundation of China [grant numbers 81770783 and 81370887].

## Conflict of Interest

The authors declare that this research was conducted in the absence of any commercial or financial relationships that could be construed as potential conflicts of interest.

## Publisher’s Note

All claims expressed in this article are solely those of the authors and do not necessarily represent those of their affiliated organizations, or those of the publisher, the editors and the reviewers. Any product that may be evaluated in this article, or claim that may be made by its manufacturer, is not guaranteed or endorsed by the publisher.

## Supplementary Material

Supplementary Figure 1 Determination of the optimal culture conditions. **(A–D)** Concentrations of IgG in the supernatant when primary B cells were treated with stimuli in a concentration gradient during the activation phase. **(E–G)** Concentrations of IgG in the supernatant when primary B cells were treated with stimuli in a concentration gradient during the differentiation period. The two-step *in vitro* B cell differentiation system was optimized by concentration gradient treatment with all stimuli. IgG secretion in the culture medium on day 12 was determined by ELISA. The error bars represent the SD. **p*<0.05 vs. 0 ng/ml group, ***p*<0.001 vs. 0 ng/ml group.

Supplementary Figure 2 Confirmation of glycosylation in IgG F(ab’)_2_ and Fc fragments. **(A)** Nonreducing SDS-PAGE gel stained with Coomassie brilliant blue, confirming that the full-length IgG molecule (left lane, 180 KD) was digested into F(ab’)_2_ (right lane, 130 KD) and the Fc fragment (right lane, 30 KD) by IdeS protease. **(B)** SNA lectin blotting was performed to confirm the glycosylation of IdeS protease-treated purified IgG. IgG obtained under control conditions was digested into F(ab’)_2_ and Fc fragments. Three biological repetitions were performed.

Supplementary Figure 3 Percentage of CD19+ B cells in peripheral blood mononuclear cells (PBMCs). **(A)** Histrogram of PBMC from healthy donor detected by flow cytometry. **(B)** Percentage of CD19+ B cells in PBMCs. The bars show the means, and the error bars show the SD.
