# Supplementary figures and images for: Cytokines in the Immune Microenvironment Change the Glycosylation of IgG by Regulating Intracellular Glycosyltransferases
Source: Front Immunol. 2022 Jan 24;12:724379. doi: 10.3389/fimmu.2021.724379 (PMC8818798; doi:10.3389/fimmu.2021.724379)

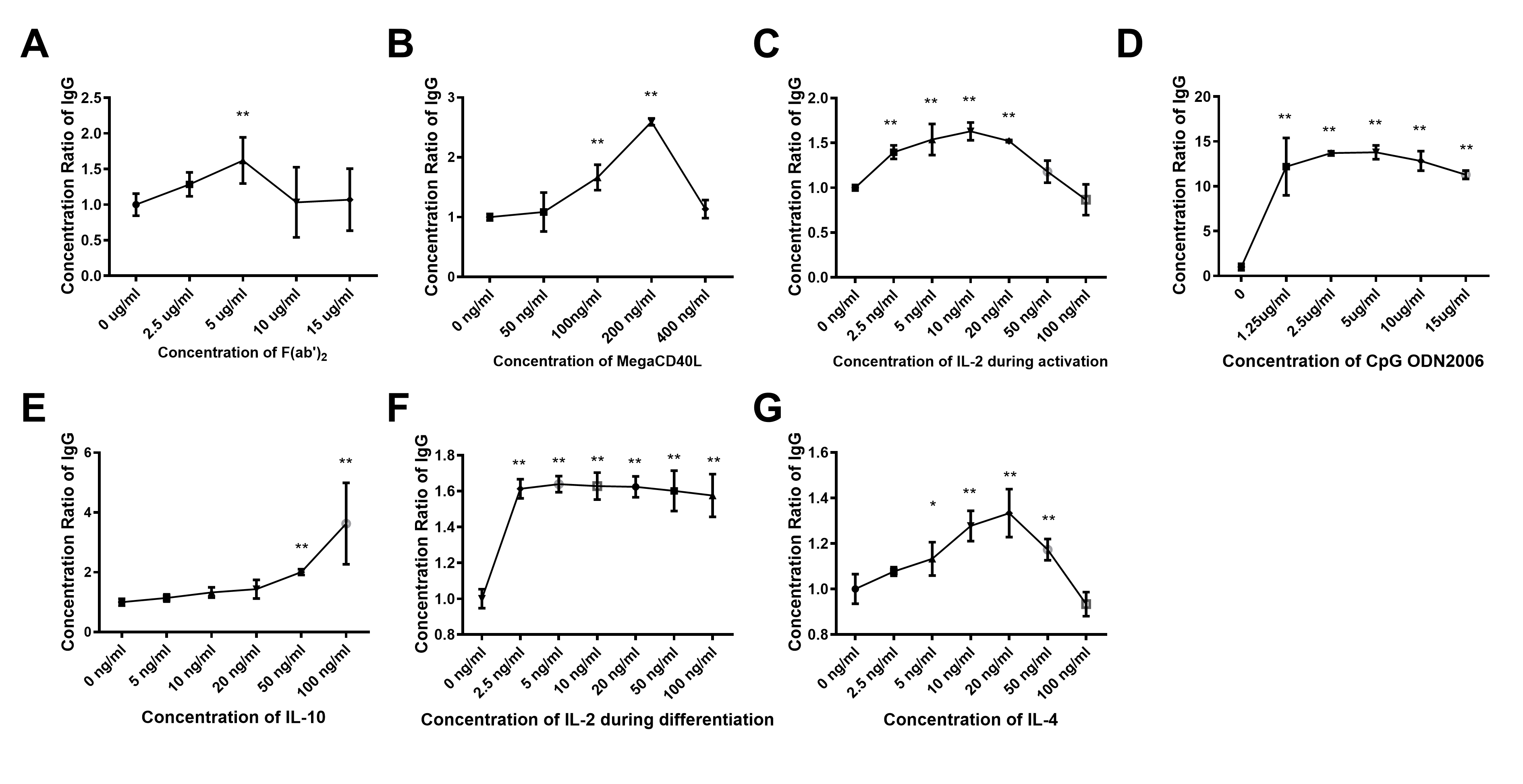

Supplement: Supplementary Figure 1 — Determination of the optimal culture conditions. (A–D) Concentrations of IgG in the supernatant when primary B cells were treated with stimuli in a concentration gradient during the activation phase. (E–G) Concentrations of IgG in the supernatant when primary B cells were treated with stimuli in a concentration gradient during the differentiation period. The two-step in vitro B cell differentiation system was optimized by concentration gradient treatment with all stimuli. IgG secretion in the culture medium on day 12 was determined by ELISA. The error bars represent the SD. *p<0.05 vs. 0 ng/ml group, **p<0.001 vs. 0 ng/ml group. [file Image_1.tif]

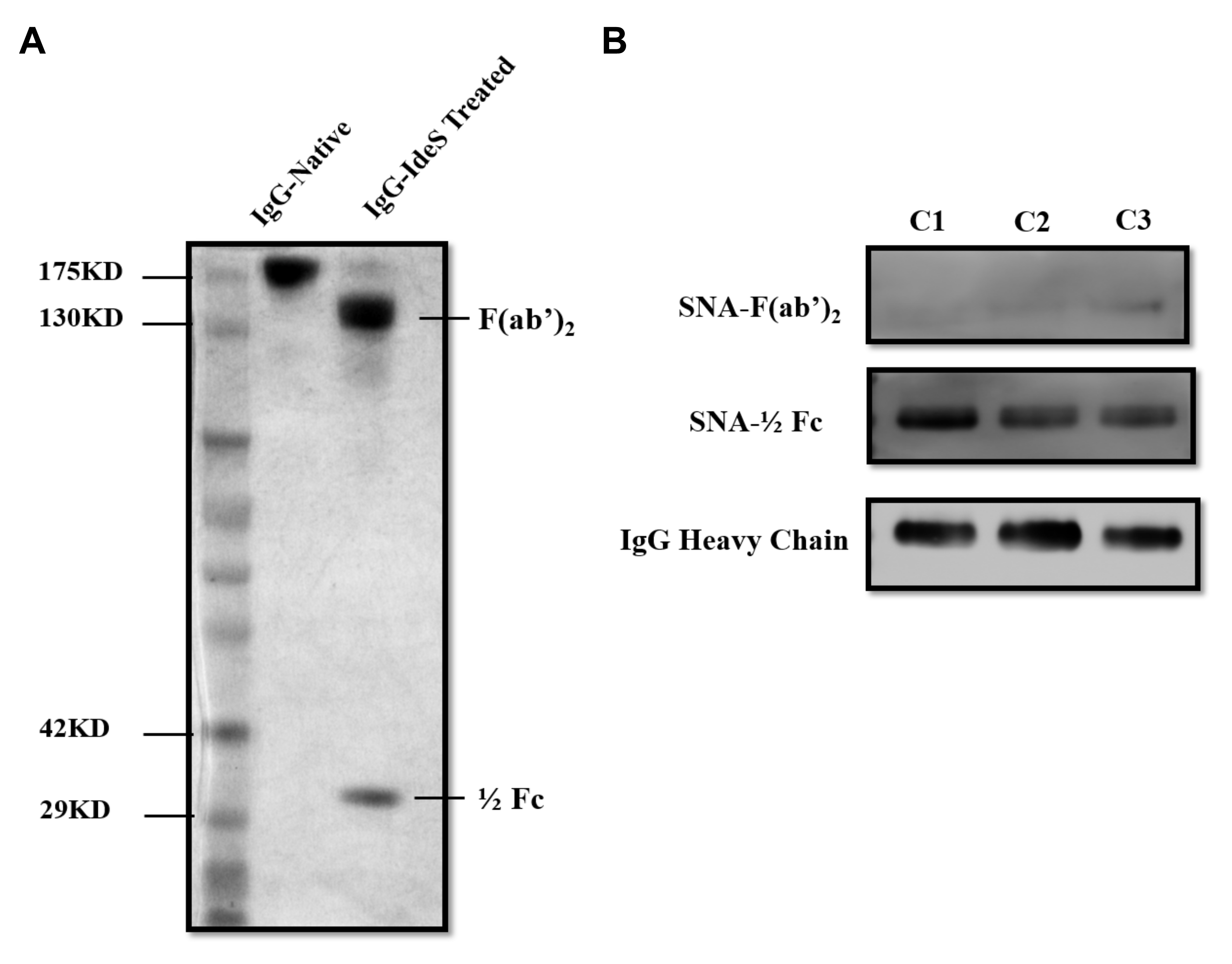

Supplement: Supplementary Figure 2 — Confirmation of glycosylation in IgG F(ab’)2 and Fc fragments. (A) Nonreducing SDS-PAGE gel stained with Coomassie brilliant blue, confirming that the full-length IgG molecule (left lane, 180 KD) was digested into F(ab’)2 (right lane, 130 KD) and the Fc fragment (right lane, 30 KD) by IdeS protease. (B) SNA lectin blotting was performed to confirm the glycosylation of IdeS protease-treated purified IgG. IgG obtained under control conditions was digested into F(ab’)2 and Fc fragments. Three biological repetitions were performed. [file Image_2.tif]

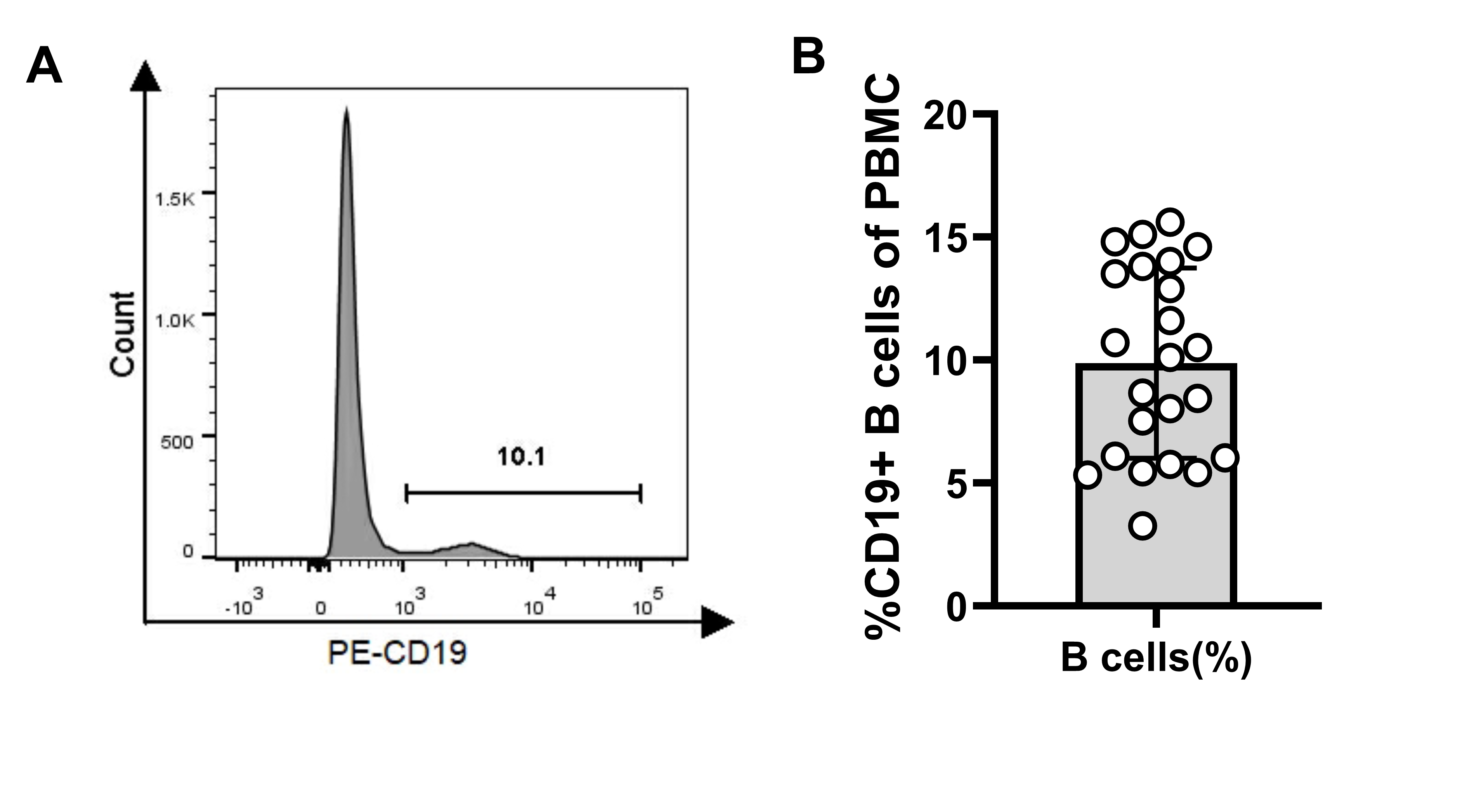

Supplement: Supplementary Figure 3 — Percentage of CD19+ B cells in peripheral blood mononuclear cells (PBMCs). (A) Histrogram of PBMC from healthy donor detected by flow cytometry. (B) Percentage of CD19+ B cells in PBMCs. The bars show the means, and the error bars show the SD. [file Image_3.tif]
