# Supplementary material for: Cytokines in the Immune Microenvironment Change the Glycosylation of IgG by Regulating Intracellular Glycosyltransferases
Source: Front Immunol. 2022 Jan 24;12:724379. doi: 10.3389/fimmu.2021.724379 (PMC8818798; doi:10.3389/fimmu.2021.724379)
Supplement: Supplementary file 5 [file Table_2.docx]

**Table S2. Calculations for derived glycosylation traits.**

| Derived trait (%) | Calculation (Relative Intensity) |
| --- | --- |
| **Fucosylated N-glycans (F)** | G0F+G1F+G0FN+G2F+G1FN+G1FS1+G2FN+G2FS1+G1FNS1+G2FNS1+G2FNS2 |
| **Agalactosylated N-glycans (G0)** | G0F+G0FN |
| **Mono-galactosylated N-glycans (G1)** | G1F+G1FN+G1FS1+G1FNS1 |
| **Digalactosylated N-glycans (G2)** | G2+G2F+G2FN+ G2FS1+G2FNS1 |
| **Asialylated N-glycans (N)** | G0F+G1F+G2+G0FN+G2F+G1FN+G2FN |
| **Mono-sialylated N-glycans (S1)** | G1FS1+G2FS1+G1FNS1+G2FNS1 |
| **Disialylated N-glycans (S2)** | G2FNS2 |
| **Bisection (B)** | G0FN+G1FN+G2FN+G1FNS1+G2FNS1+G2FNS2 |

**Table S3. Changes in glycan patterns in stimulation of cytokines compared to control group.**

| Glycan | Lectin | Preferred sugar | IFN-γ | TNF-α | IL-21 | IL-17A | APRIL |
| --- | --- | --- | --- | --- | --- | --- | --- |
| Galactose | RCA I | Gal | ^1^ |  |  |  |  |
|  | MNA-G | Galα-2,6 linked sialic acid residues |  | ^3^ |  |  |  |
|  | ECL | Galβ4GlcNAc (Terminal) |  |  |  |  |  |
|  | ABA | Galβ3GalNAc |  |  |  |  |  |
|  | PHA-L | Galβ4GlcNAcβ6 (GlcNAcβ2 Manα3) Manα3 |  |  |  |  |  |
|  | PHA-E | Galβ4GlcNAcβ2 (Galβ4GlcNAcβ6) Man |  |  |  |  |  |
|  | ACL,ACA | Galβ3GalNAc |  |  |  |  |  |
| Sialic acid | SNA-I | α-2,6 linked sialic acid residues |  |  |  |  |  |
|  | SNA,EBL | Neu5Acα6Gal/GalNAc |  |  |  |  |  |
| GlcNAc | DSL | (GlcNAc)2-4 | ^2^ |  |  |  |  |
|  | LEL,TL | GlcNAc (prefer trimer and tetramer) |  |  |  |  |  |
|  | WGA | GlcNAcβ4GlcNAc)1-4 |  |  |  |  |  |
| Mannose | MNA-M | Man |  |  |  |  |  |
|  | PSA | αMan,αGlc |  |  |  |  |  |
|  | GNL | αMan |  |  |  |  |  |

^1^ The red arrow: the ratio of S/N is significantly increased in the group compared to control group, the *p*-value<0.05.

^2^ The green arrow: the ratio of S/N is significantly decreased in the group compared to control group, the *p*-value<0.05.

^3^ The pink arrow: the ratio of S/N is tend to increase in the group compared to control group, the *p*-value<0.1

**Table S4. Enzymes involved in the process of glycosylation**

| Enzyme full name | Gene symbol | Glycan type | Identity and function |
| --- | --- | --- | --- |
| β-Galactoside α-2,6-sialyltransferase 1 | ST6GAL1 | Sialic acid | Transfers CMP-sialic acid to galactose-containing acceptor substrates. |
| Sialidase-1 | NEU1 | Sialic acid | Removes sialic acid (N-acetylneuraminic acid) moieties from complex-type N-linked oligosaccharides of glycoproteins and glycolipids. |
| β-1,4-Galactosyltransferase 1 | B4GALT1 | Galactose | Responsible for the lactose synthesis of complex-type N-linked oligosaccharides in many glycoproteins. |
| β-Galactosidase | GLB1 | Galactose | Cleaves β-linked terminal galactosyl residues from galactose-containing acceptor substrates. |

The glycosylation enzymes listed in the first column were chosen according to the glycan type with significant changes. The third column shows the glycan type with which the glycosylation enzymes act. The identity and function of each glycosylation enzyme are listed in the fourth column. The glycoform specificities of enzymes involved in the glycosylation process were summarized from the Uniprot Database (http://www.uniprot.org).
